# Small-scale field evaluation of push-pull system against early- and outdoor-biting malaria mosquitoes in an area of high pyrethroid resistance in Tanzania

**DOI:** 10.12688/wellcomeopenres.13006.1

**Published:** 2017-11-22

**Authors:** Arnold S. Mmbando, Halfan S. Ngowo, Masoud Kilalangongono, Said Abbas, Nancy S. Matowo, Sarah J. Moore, Fredros O. Okumu

**Affiliations:** 1 Environmental Health and Ecological Sciences, Ifakara Health Institute, Ifakara, Tanzania; 2 Swiss Tropical and Public Health Institute, Basel, Switzerland; 3 Univeristy of Basel, Basel, Switzerland; 4 School of Public Health, Faculty of Health Sciences, University of the Witwatersrand, Johannesburg, South Africa; 5 Institute of Biodiversity, Animal Health and Comparative Medicine, University of Glasgow, Glasgow, UK

**Keywords:** push-pull system, early-biting and outdoor-biting mosquitoes, malaria protection

## Abstract

**Background**: Despite high coverage of indoor interventions like insecticide-treated nets, mosquito-borne infections persist, partly because of outdoor-biting, early-biting and insecticide-resistant vectors. Push-pull systems, where mosquitoes are repelled from humans and attracted to nearby lethal targets, may constitute effective complementary interventions.

**Methods**: A partially randomized cross-over design was used to test efficacy of push-pull in four experimental huts and four local houses, in an area with high pyrethroid resistance in Tanzania. The push-pull system consisted of 1.1% or 2.2% w/v transfluthrin repellent dispensers and an outdoor lure-and-kill device (odour-baited mosquito landing box). Matching controls were set up without push-pull. Adult male volunteers collected mosquitoes attempting to bite them outdoors, but collections were also done indoors using exit traps in experimental huts and by volunteers in the local houses. The collections were done hourly (1830hrs-0730hrs) and mosquito catches compared between push-pull and controls. *An. gambiae* s.l. and *An. funestus* s.l. were assessed by PCR to identify sibling species, and ELISA to detect *Plasmodium falciparum* and blood meal sources.

**Results**: Push-pull in experimental huts reduced outdoor-biting for *An. arabiensis* and *Mansonia *species by 30% and 41.5% respectively. However, the reductions were marginal and insignificant for *An. funestus* (12.2%; p>0.05) and *Culex* (5%; p>0.05). Highest protection against all species occurred before 2200hrs. There was no significant difference in number of mosquitoes inside exit traps in huts with or without push-pull. In local households, push-pull significantly reduced indoor and outdoor-biting of *An. arabiensis* by 48% and 25% respectively, but had no effect on other species.

**Conclusion**: This push-pull system offered modest protection against outdoor-biting *An. arabiensis*, without increasing indoor mosquito densities. Additional experimentation is required to assess how transfluthrin-based products affect mosquito blood-feeding and mortality in push-pull contexts. This approach, if optimised, could potentially complement existing malaria interventions even in areas with high pyrethroid resistance.

## Introduction

Preventing human exposure to infectious mosquitoes is a crucial approach towards controlling the spread of mosquito-borne infections in Africa. Between 2000 and 2015, insecticide-treated nets (ITNs) and indoor residual spraying (IRS) reduced malaria prevalence by 68% and 10%, respectively, among 2–10 year-olds in Africa ^1^. In Tanzania, there was a national decline of malaria prevalence from 10% in 2008 to 9.5% in 2012, mostly due to widespread use of ITNs and IRS, but also improved treatments and diagnosis of the disease. However, there appears that there has been a minor resurgence in malaria, according to a current national prevalence report, stated at 14.8% ^2^. It is expected that ongoing efforts, including the most recent LLIN universal coverage campaign completed in 2016, will improve the situation.

Despite the improved malaria vector control investments in recent years, endemic countries still face various challenges. Examples include the increasing outdoor-biting and early-biting mosquito behaviours, which limit efficacy of ITNs and IRS, both of which primarily target indoor-biting mosquitoes ^3, 4^. Another challenge is the widespread insecticide resistance in major vector populations ^5– 7^. To address early-biting, outdoor-biting and insecticide-resistant mosquitoes, there is a need for simple and low-cost approaches applicable even in rural and remote areas. Fortunately, there have been many recent advances and several promising new products have been developed that aim to reduce outdoor-biting, which could be optimized ^8^. These include area-wide mosquito repellents, also called spatial repellents, such as transfluthrin-treated materials ^9^, and odour-baited mosquito-control devices, such as mosquito landing box (MLB), which have been demonstrated to reduce vector densities and survival ^10, 11^. Related interventions that may also mitigate insecticide resistance include traps with electrocuting grids ^10^, mosquito-killing fungi, such as *Metarhizium anisopliae* ^12^, and combination of insecticides with different modes of action ^13^. Personal protection with repellents also prevents outdoor-bites but are affected by poor compliance among users ^14^. In some circumstances, repellents do prevent outdoor bites but may divert mosquitoes from protected to unprotected individuals ^15, 16^.

Push-pull systems could be another solution against early-biting, outdoor-biting and resistant vectors, by repelling host-seeking mosquitoes from humans and luring them towards killing stations, using species-specific lures. Such approaches have been used effectively in integrated pest management in agriculture, where behaviour-modifying stimuli are deployed to manipulate and reduce pest populations ^17– 19^. Similar systems were demonstrated to reduce malaria vector biting by 95% under controlled semi-field conditions in Western Kenya ^20^.

We conducted a small-scale field experiment to assess the efficacy of a simple push-pull strategy, consisting of evaporated transfluthrin and odour-baited lure-and-kill stations, all set in peri-domestic spaces, against early-biting, outdoor-biting pyrethroid-resistant malaria mosquitoes.

## Methods

### Study area

**Figure 1.**
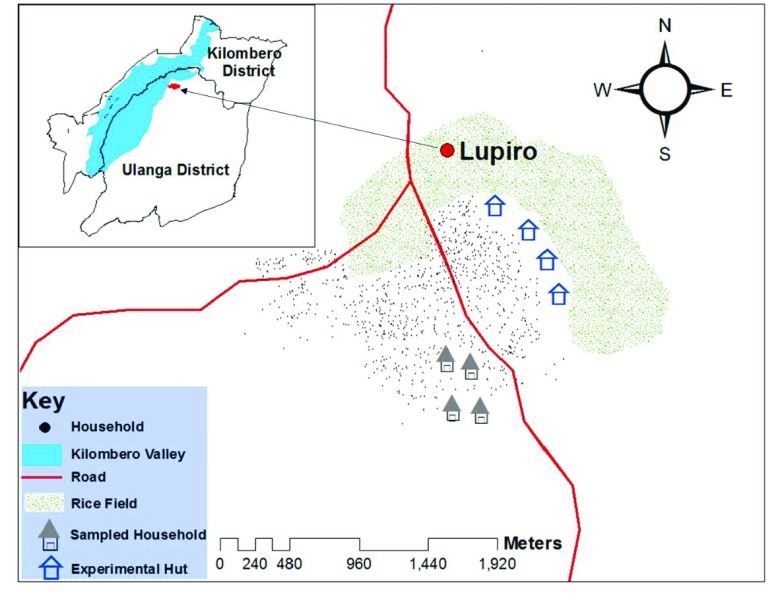
A map of the study area (Lupiro village in the Ulanga district, south-eastern Tanzania). Experimental huts and local houses used to test efficacy of push-pull are shown.

The study was conducted in Lupiro village, in the plains of the Kilombero valley, approximately 30km from Ifakara town, in south-eastern Tanzania ( Figure 1). This village has mesoendemic malaria transmission, mediated primarily by *An. funestus* s.s. that bite indoors ^15^ and *An*. *arabiensis,* which occurs in larger numbers and bites people from early-evening both outdoors and indoors before and during bed time ^21^. The main malaria control intervention used in the study area is long lasting insecticidal treated nets (LLINs) ^22^. Recent studies conducted in 2016 have confirmed widespread pyrethroid resistance in the area in both *An. arabiensis* ^23^, and *An. funestus* mosquitoes ^24^.

### Field-testing the efficacy of push-pull in specially designed experimental huts

A partially randomized crossover study was done in four experimental huts, to assess field efficacy of push-pull over 32 nights. The system consisted of an effective spatial repellent (transfluthrin), and an odour-baited lure-and-kill device (mosquito landing box (MLB)), fitted with low-cost electrocuting grids ^10^. The transfluthrin was dispersed from ten polyester strips measuring 1 × 25cm, which were cut out of untreated polyester net (Safi net, A to Z Textile Mills Ltd, Arusha, Tanzania), as recently described ^25^. These strips were then soaked in 1.1% of transfluthrin stock solution diluted in ethanol for 30 minutes and then suspended in the odour-dispensing section of the modified MLB ^25^. By contrast, the odour-baited MLB was baited with a synthetic lure (i.e. 4-compound Ifakara blend formulated by Biogents, Germany) ^26^ together with carbon dioxide gas from yeast-molasses fermentation ^11^.

**Figure 2.**
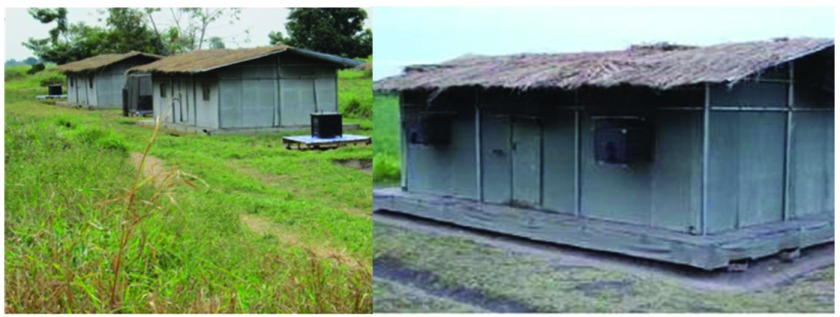
Ifakara experimental hut ^27^. These are single-room model huts for entomological studies. They have eave spaces to allow mosquito entry and can be fitted with interception traps on these eaves and windows to collect mosquitoes as they exit from the hut.

**Figure 3.**
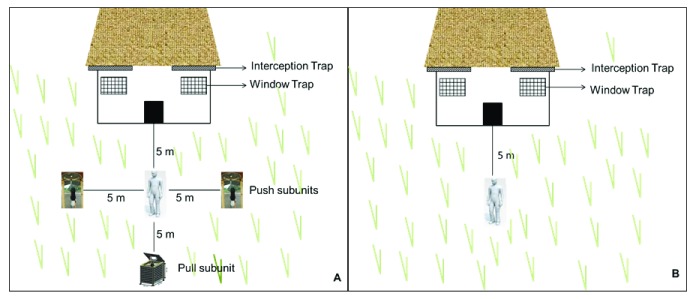
Illustration of the push-pull strategy as tested in this study. The test configuration consisted of two push sub units (spatial repellent dispensers) and one pull sub unit (an attractant-baited mosquito control device). ( **A** and **B**) show treatment and control settings around experimental huts. Host-seeking mosquitoes repelled from around human dwellings are lured then killed by the odour-baited device. The configuration, physical location of sub units, and distances between the units were only assumed, and had not been previously tested, but was considered representative of likely use scenarios.

Two experimental huts were used as treatment (i.e. having the push-pull system ( Figure 2)), and another two huts as controls (i.e. without push-pull). These huts were modelled on local houses in the study area, and have been proven to be effective for monitoring natural behaviours of wild mosquito populations ^27^. The huts were fitted with exit interception traps on eaves and windows to collect all mosquitoes that had entered the huts. The configuration of the push-pull sub units, including physical location near the huts and distances between sub units was only assumed, but not previously tested, yet representative of likely use scenarios ( Figure 3A).

In this study, the huts were 50m apart to reduce any interactions between treatments. Two repellent active emanators (push) were set 5m away from the pull device outside the treatment huts. One volunteer sat between push and pull subunits, and performed human landing catches (HLC) ^28^. This volunteer was close to where people would normally be sitting in the evening, which was within 5m of the hut. A second person was located indoors under an untreated bed net to act as bait. The pull device (i.e. the MLB) was placed 10m away from the hut, but approximately 8m from push subunits (repellent dispensers) and a volunteer sitting in the peri-domestic space conducting HLC. Mosquitoes were also collected in the exit traps in the huts. Control huts also had volunteers but no push-pull units ( Figure 3B). All mosquito collections were done between 1800 and 0700 hours, for 30 minutes each hour. The volunteer pairs were rotated nightly between the control and treatment huts to minimize any bias due to difference in individual attractiveness. The experiment was conducted over 16 nights, working 4 nights/week, then the intervention and control huts were interchanged, and the test repeated for another 16 nights.

The main outcome measure in this experiment was number of mosquitoes of different species attempting to bite humans outdoors, and number collected indoors in the exit traps. We also assessed differences of indoor catches between treatment and control huts to estimate diversion due to the push-pull subunits outdoors.

### Field-testing the efficacy of push-pull in local households

This activity was similar to first one, but was conducted in actual households in the study village. Four houses, at least 100m apart were selected and the experiment done for 16 nights, working 4 nights per week. Two of the houses were allocated as treatment (with push-pull subunits) and the other two were controls (without push-pull subunits). The four houses had two occupants, four open eave spaces, two screened windows, and corrugated iron roof. The mosquito sampling rooms had one window, approximately 1m ^2^, with walls. There were two male volunteers assigned per house: one was conducting HLC inside and the other outside for 30minutes each hour. The volunteer pairs were rotated between treatment and control houses.

The following changes were introduced based on lessons from the first experiment: 1) amount of transfluthrin was doubled to 2.3% w/v (180mg transfluthrin dissolved into 8ml 70% ethanol) to increase repellence strength; 2) distance between the odour-baited MLB and human was increased to 10m to reduce the possibility of the MLB increasing mosquitoes close to human volunteer; and 3) CO _2_ from yeast-sugar fermentation was replaced with industrial CO _2_ at 230ml/min, thereby ensuring consistent flow throughout the night. Similar to the first experiment, the main outcome measure was number of mosquitoes of different species attempting to bite humans outdoors, and number collected indoors in the exit traps.

### Laboratory analysis of sampled malaria vectors to distinguish between species

Mosquitoes collected from the two experiments were morphologically identified and grouped by taxa, sex and physiological state (blood fed, unfed or gravid). Female *An. gambiae* s.l. complex and *An. funestus* group were packed in batches of 10 mosquitoes, in labelled plastic micro-centrifuge tubes (Eppendorf ^®^), containing pellets silica (desicant), with cotton plugs to avoid damaging mosquito carcases.

*An. gambiae s.l.* and *An. funestus s.l* were further analysed by Polymerase Chain Reaction (PCR) to distinguish between sibling species. Mosquito genomic DNA of *An. gambiae* s.l was extracted from the two adult mosquito hind legs, as previously described by Scott *et al.* ^29^. The PCR amplification was based on the species-specific nucleotide sequence of the ribosomal DNA (rDNA) intergenic spacer regions (IGS). The IGS region of the rDNA was amplified in a 25µl reaction volume of PCR mixture following Scott *et al.* protocol ^29^. For the *An. funestus* group, we used methods developed by Koekemoer *et al.* ^30^, to detect five members of the *An. funestus* group. This rDNA-PCR amplification was based on the species-specific primers in the non-coding region called Internal Transcribed Spacer 2 (ITS2) on the rDNA ^30^. Post-PCR amplicons from *An. gambiae* and *An. funestus* PCR assays were analysed by electrophoresis in agarose gel stained with ethidium bromide. Visible DNA bands were photographed under ultraviolet light using Kodack Gel Logic 100 imaging system.

### Laboratory analysis of sampled malaria vectors to detect malaria parasite infections

Enzyme linked immunosorbent assays (ELISA) for detection of *Plasmodium falciparum* circumsporozoite protein in the salivary glands of the pooled *Anopheles* samples ^31^. Detection of *P. falciparum* parasites were performed from all caught malaria vectors *An. arabiensis* and *An. funestus.* The optical density of post-ELISA lysate were measured at 405 - 414nm after 45 minutes using ELISA plate reader machine ^31^.

### Laboratory analysis of sampled malaria vectors to identify sources of mosquito blood meals

ELISA assays were also done for determination of sources of mosquito blood meals by using abdomen of blood-fed mosquitoes caught in the study. The ELISA method was used to detect host-blood antigens found in abdomens of blood-fed mosquitoes ^32^, assessing presence of human, bovine, goat, dog and chicken blood. Anti-IgG antibodies from each host were used to detect host antigen in the blood meal of the mosquito. Similar measurements of optical density of post-ELISA lysates were performed as in the circumsporozoite ELISA technique.

### Data analysis

Analysis and power calculations were performed using R statistical software ^33^. The power calculations were performed before starting the experiment through statistical simulations using data from previous studies conducted by Ifakara Health Institute (IHI). Using an approach developed by Johnson *et al.* ^34^, simulations with 30 mosquito landings per night, 32 replicates in a 4×4 Latin square experiment and a hypothesised protective efficacy of 30%, resulted in 82% (Confidence Interval; 79% - 84%) power at 95% confidence.

The R version 3.3.2 package *lme4* was used to perform generalized linear mixed effects modelling (GLMM) and to examine the protective efficacy of the push-pull system and its characteristics on mosquito catches ^35^. Since the data were highly left-skewed, negative binomial distributions with log-link functions were used to model the data. Mosquito catches were modelled as a function of fixed factors: 1) volunteer positions (inside or outside) and treatment (with or without push-pull), combined as a single variable; 2) location; and 3) volunteer pairs. Sampling night and experimental rounds were included as random variables to account for natural heterogeneity in the data. Mosquito hourly catches (only females) were pooled by species for each nightly catches. Four interactions were created: Interaction-1 (control*outdoor), Interaction-2 (control*indoor), Interaction-3 (push-pull*indoor) and Interaction-4 (push-pull*outdoor). Interaction-1 was used as reference for measuring mosquito bite prevention between controls outdoor against push-pull outdoor, while interaction-2 was used as reference for measuring mosquito diversion from outdoors where push-pull subunits were towards indoors in the treatment sites compared to controls.

Mosquito hourly data were summarized into cumulative catches caught at specified night periods, early night (1900–2200 hours), midnight period (2300–0400 hours) and early morning (0500–0700 hours). The hourly data were also pooled to obtain nightly average mosquito densities for each location and treatment. Laboratory mosquito data were counted, and summarized using descriptive statistics by calculating proportions and percentage of mosquito species identified, proportions with different host blood meal and sporozoite ELISA detection rates.

### Ethics statement

Volunteers participating in the study were adequately informed of the study objectives, potential benefits and potential risks, after which written informed consent was obtained. Adequate training on experimental procedures was given to the volunteers. Chemoprophylaxis and medical supervision was also offered by trained clinical officer, according to Tanzania guidelines on prevention and treatment of malaria ^36^. No volunteer fell ill during the experiments. Ethical approval was obtained from the Institutional Review Board of Ifakara Health Institute (IHI/IRB/EXT/09-2017) and the Medical Research Coordinating Council at Tanzanian National Institute of Medical Research (NIMR/HQ/R.8a/Vol.IX/2199). Permission to publish this work was also obtained from the National Institute for Medical Research (NIMR, reference number NIMR/HQ/P.12 VOL.XXII/9). Reprints and online links to this work will be provided to NIMR after publication.

## Results

### Field-testing the efficacy of push-pull in specially designed experimental huts

There were moderate reductions in landings by host-seeking malaria vectors and non-malaria vectors on volunteers sitting beside treatment huts (with push-pull) compared to control huts (without push-pull). The push-pull system significantly reduced *An. arabiensis* landings by 30% (incidence rate ratio (IRR) = 0.70 (0.56 - 0.87), P < 0.001), and *Mansonia species* landings by 41.5% (IRR= 0.59 (0.51 - 1.35), P = 0.014). There was a non-significant 12.2% reduction of *An. funestus* landings (IRR= 0.88 (0.72 -1.09), P = 0.256). Nuisance biting *Culex* mosquito landings were not reduced (IRR= 0.95 (0.79 -1.14), P = 0.584) ( Table 1).

**Table 1.** Number of host-seeking mosquitoes caught attempting to bite volunteers outdoors in the treatment experimental huts (with push-pull) and control huts (without push-pull).

Variables	Descriptions	N ^n^	∑ ^mosq^	% Protection	Median	IQR	IRR	95% CI around IRR	P value
*An. gambiae* complex									
Bite prevention	Control outdoors	64	2393	1	28	(15 – 53.25)	1	-	-
	Push-pull outdoors	64	1650	0.31	21.5	(11 – 37.25)	0.700	(0.566 - 0.866)	< 0.001
	Control indoors	64	1207	1	12.5	(7 - 27.25)	0.466	(0.375 - 0.578)	< 0.001
	Push-pull indoors	64	986	0.19	12	(6.75 - 20.25)	0.413	(0.332 - 0.514)	< 0.001
*An. funestus group*									
Bite prevention	Control outdoors	64	464	1	5	(3 – 10)	1	-	-
	Push-pull outdoors	64	396	0.15	5	(3 – 9)	0.888	(0.724 - 1.090)	0.256
	Control indoors	64	782	1	10	(6 – 16)	1.719	(1.418 - 2.084)	< 0.001
	Push-pull indoors	64	726	0.07	11	(7 – 16)	1.641	(1.353 - 1.991)	< 0.001
*Mansonia species*									
Bite prevention	Control outdoors	64	1528	1	14.5	(6 – 28.25)	1	-	-
	Push-pull outdoors	64	876	0.43	9	(3 – 24.5)	0.595	(0.393 - 0.901)	0.0143
	Control indoors	64	274	1	1	(0 – 3)	1.114	(0.072 - 0.182)	< 0.001
	Push-pull indoors	64	214	0.22	0	(0 – 3)	0.094	(0.060 - 0.149)	< 0.001
*Culex* species									
Bite prevention	Control outdoors	64	3915	1	52	(33 – 71.5)	1	-	-
	Push-pull outdoors	64	3629	0.07	48	(30.75 – 71.00)	0.950	(0.790 - 1.142)	0.584
	Control indoors	64	8723	1	138	(97.75 – 172.50)	2.328	(1.940 - 2.793)	< 0.001
	Push-pull indoors	64	8452	0.03	122	(92.25 – 172.50)	2.279	(1.898 - 2.735)	< 0.001

** % Protection = ∑ ^mosq^ caught in (Control) - Push-pull (treatment))/Control.

Push-pull did not increase indoor densities in treated houses, as would be expected if diversion were occurring. In fact, the treatment slightly reduced mosquito entry into the treated huts, though these increases were not statistically significant for any ( Table 2). We observed 11.3% (IRR= 0.88 (0.711 - 1.08), P = 0.291) reduction in *An. arabiensis*, 5.5% (IRR= 0.955 (0.79 – 1.15), P = 0.691) reduction in *An. funestus* s.s., 17.4% (IRR= 0.83 (0.51 - 1.35), P = 0.446) reduction in *Mansonia species* and 2.3% (IRR= 0.98 (0.82 – 1.17), P = 0.815) reduction in *Culex* mosquitoes ( Table 2). Data also showed a higher proportion of *An. funestus* host-seeking mosquitoes were caught indoors than outdoors, which was opposite for *An. arabiensis*, for which catches were higher outdoors.

**Table 2.** Number of host seeking-seeking mosquitoes caught indoors in treated experimental huts (with push-pull) and control huts (without push-pull).

Variables	Descriptions	N ^n^	∑ ^mosq^	% Protection	Median	IQR	IRR	95% CI around IRR	P value
*An. gambiae* complex									
Diversion effect	Control indoors	64	1207	1	12.5	(7 - 27.25)	1	-	-
	Push-pull indoors	64	986	0.19	12	(6.75 - 20.25)	0.887	(0.711 - 1.08)	0.291
	Control outdoors	64	2393	1	28	(15 – 53.25)	2.147	(1.730 - 2.664)	< 0.001
	Push-pull outdoors	64	1650	0.31	21.5	(11 – 37.25)	1.503	(1.209 - 1.868)	< 0.001
*An. funestus group*									
Diversion effect	Control indoors	64	782	1	10	(6 – 16)	1	-	-
	Push-pull indoors	64	726	0.07	11	(7 – 16)	0.955	(0.794 - 1.147)	0.619
	Control outdoors	64	464	1	5	(3 – 10)	0.582	(0.480 - 0.705)	< 0.001
	Push-pull outdoors	64	396	0.15	5	(3 – 9)	0.517	(0.425 - 0.628)	< 0.001
*Mansonia species*									
Diversion effect	Control indoors	64	274	1	1	(0 – 3)	1	-	-
	Push-pull indoors	64	214	0.22	0	(0 – 3)	0.826	(0.505 - 1.351)	0.446
	Control outdoors	64	1528	1	14.5	(6 – 28.25)	8.755	(5.502 - 13.932)	< 0.001
	Push-pull outdoors	64	876	0.43	9	(3 – 24.5)	5.210	(3.278 - 8.280)	< 0.001
*Culex species*									
Diversion effect	Control indoors	64	8723	1	138	(97.75 – 172.50)	1	-	-
	Push-pull indoors	64	8452	0.03	122	(92.25 – 172.50)	0.979	(0.818 - 1.171)	0.815
	Control outdoors	64	3915	1	52	(33 – 71.5)	0.430	(0.358 - 0.515)	< 0.001
	Push-pull outdoors	64	3929	0.07	48	(30.75 – 71.00)	0.408	(0.340 - 0.489)	< 0.001

**Mosquito diversion effect = Number of mosquito caught indoor and outdoor in control vs. treatment hut

**Figure 4.**
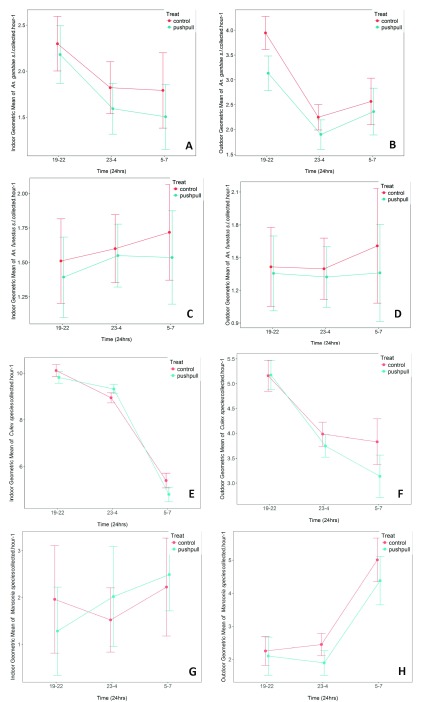
Cumulative geometric mean number of host-seeking malaria vectors caught indoors and outdoors. ( **A** & **B**) represent *Anopheles gambiae*; ( **C** & **D**) represent *Anopheles funestus*; ( **E** & **F**) represent *Culex* species; ( **G** & **H**) represent *Mansonia species.* The mosquitoes were caught during early night, midnight and early morning hours. Differences between treated and control huts are shown. Error bars represent 95% Confidence Intervals.

Push-pull showed only marginal protection in the early night period (1900 – 2200 hours) against host-seeking malaria and non-malaria vectors caught outdoors and indoors ( Figure 4). During early night hours, it reduced *An. arabiensis* landings by 20.5% (geometric mean (GM) of (3.1 (2.8 – 3.5)) in treatment compared to (3.9 (3.6 – 4.2)) in controls). There was no effect on *Culex* mosquitoes and no outdoor protection ( Figure 4E & F)

### Field-testing the efficacy of push-pull in local households

The push-pull system significantly reduced *An. arabiensis* landings by 25% (IRR= 0.75 (0.53 – 0.98), P =0.0024), but only marginally reduced *Culex* landings, i.e. by 16% (IRR= 0.84 (0.58 – 1.16), P =0.467). The system also did not offer any protection to human volunteers against *An. funestus* or *Mansonia* mosquito bites at both indoor and outdoor locations. There was a non-significant increase in outdoor landings for *An. funestus* (IRR=1.55 (0.55 - 4.28), P=0.678) and an increase in *Mansonia species* landings (IRR= 1.12 (0.25 - 4.81), P =0.345) at the treatment houses compared to control ( Table 3). This data however remains inconclusive as the densities for both *An. funestus* and *Mansonia species* were very low.

**Table 3.** Number of host-seeking mosquitoes caught attempting to bite volunteers outdoors in local households with push-pull and control households.

Variables	Descriptions	N ^n^	∑ ^mosq^	% Protection	Median	IQR	IRR	95% CI around IRR	P value
*An. gambiae* complex									
Bite prevention	Control outdoors	32	544	1	16	(10 - 23)	1	-	
	Push-pull outdoors	32	404	0.26	9	(6 – 17)	0.75	(0.55 – 1.17)	0.0024
	Control indoors	32	164	1	4	(2.75 - 6)	0.30	(0.22 – 0.43)	<0.001
	Push-pull indoors	32	83	0.49	2	(0.75 - 3.25)	0.16	(0.11 – 0.22)	<0.001
*An. funestus group*									
Bite prevention	Control outdoors	32	9	1	0	(0 – 1)	1	-	
	Push-pull outdoors	32	9	0	0.5	(0 – 1.25)	1.55	(0.56 – 4.33)	0.678
	Control indoors	32	13	1	0	(0 – 1)	2.19	(0.84 - 5.71)	>0.05
	Push-pull indoors	32	25	-0.48	0	(0 – 0)	4.31	(1.76 – 10.44)	<0.05
*Mansonia species*									
Bite prevention	Control outdoors	32	10	1	0	(0 – 0)	1	-	
	Push-pull outdoors	32	11	-0.1	0	(0 – 0.25)	1.12	(0.26 - 4.90)	0.345
	Control indoors	32	5	1	0	(0 – 0)	0.52	(0.10 - 3.01)	>0.05
	Push-pull indoors	32	21	-3.2	0	(0 – 0)	2.09	(0.49 - 8.82)	>0.05
*Culex species*									
Bite prevention	Control outdoors	32	429	1	12.5	(5 – 21.25)	1	-	
	Push-pull outdoors	32	330	0.23	9.5	(4 – 12.25)	0.84	(0.59 - 1.20)	0.467
	Control indoors	32	920	1	21	(6.75 – 41.25)	1.86	(1.31 – 2.64)	0.234
	Push-pull indoors	32	900	0.02	17	(12 – 44.5)	2.04	(1.44 – 2.88)	<0.01

** % Protection = ∑ ^mosq^ caught in (Control - Push-pull (treatment))/Control.

Presence of the push-pull system in the peri-domestic space significantly reduced indoor densities for *An. arabiensis* mosquitoes by 48% (IRR=0.52 (0.35 – 0.76), P= 0.006). The data on *An. funestus* and *Mansonia species* was however inconclusive as densities for these two species were very low ( Table 4).

**Table 4.** Number of host-seeking mosquitoes caught indoors in local households with push-pull and control households.

Variables	Descriptions	N ^n^	∑ ^mosq^	% Protection	Median	IQR	IRR	95% CI around IRR	P value
*An. gambiae* complex									
Diversion effect	Control indoors	32	164	1	4	(2.75 – 6)	1	-	
	Push-pull indoors	32	83	0.49	2	(0.75 – 3.25)	0.52	(0.35 – 0.76)	0.006
	Control outdoors	32	544	1	16	(10 – 23)	3.25	(2.33 – 4.53)	< 0.001
	Push-pull outdoors	32	404	0.26	9	(6 – 17)	2.42	(1.72 – 3.40)	< 0.001
*An. funestus group*									
Diversion effect	Control indoors	32	13	1	0	(0 – 1)	1	-	
	Push-pull indoors	32	25	-0.48	0	(0 – 0)	2.07	(1.00 – 3.86)	0.02
	Control outdoors	32	9	1	0	(0 – 1)	0.46	(0.18 – 1.21)	> 0.05
	Push-pull outdoors	32	9	0	0.5	(0 – 1.25)	0.71	(0.30 – 1.66)	> 0.05
*Mansonia species*									
	Control indoors	32	5	1	0	(0 – 0)	1	-	
	Push-pull indoors	32	21	-3.2	0	(0 – 0)	4.01	(0.82 – 20.0)	0.458
	Control outdoors	32	10	1	0	(0 – 0)	1.98	(0.39 – 9.99)	> 0.05
	Push-pull outdoors	32	11	-0.1	0	(0 – 0.25)	2.16	(0.44 – 10.85)	> 0.05
*Culex species*									
Diversion effect	Control indoors	32	920	1	21	(6.75 – 41.25)	1	-	
	Push-pull indoors	32	900	0.02	17	(12 – 44.5)	1.10	(0.79 – 1.53)	0.134
	Control outdoors	32	429	1	12.5	(5 – 21.25)	0.54	(0.40 – 0.79)	< 0.05
	Push-pull outdoors	32	330	0.23	9.5	(4 – 12.25)	0.45	(0.32 – 0.65)	< 0.001

**Mosquito diversion effect = Number of mosquito caught indoor and outdoor in control vs. treatment households

**Figure 5.**
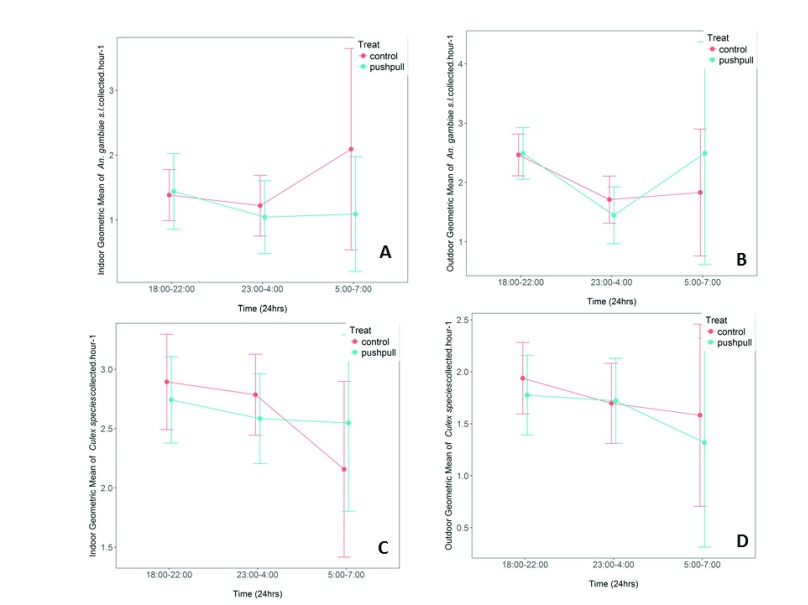
Cumulative geometric mean number of host-seeking malaria vectors and non-malaria vectors caught indoors and outdoors. ( **A** & **B**) represent *An. gambiae*; ( **C** & **D**) represent *Culex species*. The mosquitoes were caught during early night, midnight and early morning hours. Differences between treated and control houses are shown. Error bars represent 95% Confidence Intervals.

When data was segregated by period of night, we observed that push-pull systems elicited only a small magnitude of protection against host-seeking mosquito bites at specific periods of night (early night, midnight and early morning). For *An. arabiensis* mosquitoes, no protective effect was seen early in the night (1830 – 2200 hours) either indoors or outdoors, but a small protection was seen during midnight (2300 – 0400 hours) ( Figure 5). Effects on all species are shown in Table 3 and Table 4.

### Sibling species of major *Anopheles* mosquitoes, parasite infection rates and main sources of blood meals

We obtained 1247 successful PCR amplifications out of the 1385 *An. gambiae* s.l analysed by PCR. All of these were determined as *An. arabiensis*. For the *An. funestus* group, there were 1776 successful PCR amplifications, among which three sibling species as follows: 86.9% (1545 /1776) were *An. funestus* sensu stricto, 9% (160/1776) were *An. rivulorum,* and 4% (71/1776) were *An. leesoni*.

The main malaria vectors mosquitoes, *An. funestus* group (6,236 samples analysed in pools of 10 mosquitoes each) and *An. gambiae* s.l (2,368 samples analysed in pools of 10), caught were also subjected to *P. falciparum* circumsporozoites ELISA, but none of these was found infected.

A total of 47 blood-fed *An. funestus* s.l. mosquitoes were caught inside the experimental huts by using exit traps, 40 of which were *An. funestus s.s*, five being *An. rivulorum* and the remaining two being *An. leesoni*. All the blood-fed *An. funestus* s.s were confirmed by ELISA to have human blood. Of the five blood-fed *An. rivulorum* mosquitoes, four (80%) had human blood and the rest had dog-blood. The two blood-fed *An. leesoni* both had dog blood.

## Discussion

This study assessed the efficacy of a simple push-pull strategy, consisting of evaporated transfluthrin and odour-baited lure-and-kill stations in peri-domestic spaces, against early-biting, outdoor-biting pyrethroid resistant malaria mosquitoes. Neither the placement of the push-pull subunits, the distances between these units nor the distances from the individual experimental houses had been previously tested. Instead, we selected a configuration most representative of expected use cases.

Overall, this study demonstrated that the push-pull system, in the configuration tested here, reduced host-seeking mosquito landings on volunteers sitting outdoors, without increasing any biting risk indoors. Most of the protection was observed against *An. arabiensis*, and there was a very minimal effect against *An. funestus,* which recently has been shown to be the most dominant malaria vector in the study area, despite occurring in relatively low numbers ^21^. The selection of candidate attractants and repellents was based on evidence from previous studies on efficacy of various candidates. For example, an earlier study in Tanzania demonstrated that transfluthrin can prevent > 80% of mosquito bites over 20 metres (medium range) by actively emanating a repellent from an odour-dispenser section of the existing odour-baited mosquito landing box ^25^. By combining this active repellent dispenser with affordable lure-and-kill technologies ^10, 11^, we intended to create a simple push-pull system offering peri-domestic protection to complement existing strategies, such as LLINs. Though the efficacy was only modest, it is an important outcome, given the study area was characterised by widespread pyrethroid resistance ^24^. Indeed, stimuli-diversionary approaches, such as tested here, could potentially slow the spread of insecticide resistance, since they have two components that concurrently target vectors differently.

The efficacy of push-pull system was greater against *An. arabiensis* compared to *An. funestus* landings. A similar observation had been seen in previous studies when either push or pull subunits was tested separately against these mosquito species ^16, 25^. Paliga *et al.* has also recently demonstrated marginal effects of transfluthrin on *An. funestus* in a study where *An. arabiensis* were significantly repelled ^37^. This suggests that this mosquito species may be unresponsive to the repellent effects of transfluthrin at the doses used in these studies. Though we did not investigate potential causes of these differential effects on the two species, we hypothesise that it may have been due to differences in levels of insecticide resistance in these species, and differences in feeding and resting behaviours exhibited by these two mosquito species made them respond differently to the push subunits. For example, *An. funestus* are highly anthropophilic and endophilic vectors ^38^. Together with their resistance to pyrethroids (which potentially confers cross-resistance to transfluthrin), this could make the mosquitoes still bite humans despite airborne transfluthrin. This was not the case for *An. arabiensis,* which exhibit a wide range of behavioural responses, both biting and resting, making them avoid treated areas and chose different hosts when humans are protected ^4, 39^. Both feeding and resting behaviours might be the reason, which also made these mosquitoes respond differently with our push-pull system. It is well known that less anthropophilic mosquitoes are repelled at lower doses of repellents than highly anthropophilic vectors ^40^.

In previous semi-field tests, the MLB fitted with low-cost electrocuting grids situated at medium range from a human volunteer successfully reduced outdoor densities of host-seeking *An. arabiensis* ^10^. The modest biting protection offered by our push-pull system against primary malaria vectors is very crucial, especially in rural villages where people spend early night period outdoor and indoor conducting various activities. These times coincide with most domestic activities, such as cooking, washing dishes, and storytelling outdoors ^41^. The system also reduced indoor densities and outdoor nuisance bites of *Culex* and *Mansonia* mosquitoes; hence there could be potential against arbovirus vectors or nuisance mosquitoes. Prevention of nuisance bites is also essential because it increases compliance with an intervention if users perceive a benefit ^42^.

When data were analysed to depict nightly patterns, there was a slight reduction of bites from different mosquito species caught in each night periods, early night, midnight and early morning. There were a high number of mosquitoes caught in early night hours (1900 to 2200 hours) when humans are often active either indoors or outdoors. The protective efficacy of the system during early night outdoors was better seen against malaria vectors than for non-malaria mosquitoes. This protection is important because the system offered protection to people before they sleep under the bed net, thus covering mosquito-bite protection gap against early-biting, outdoor-biting species. From a personal protection point of view, it is this time of night when the complementary value of the push-pull system is most relevant. Early-biting and outdoor-biting mosquitoes, which remain a major challenge to malaria control ^38^, can be controlled by using many additional interventions including push-pull system to target these subpopulations.

During this small-scale field evaluation of the efficacy of the push-pull system in local household settings, mosquito hourly data were summarized into nightly catches as in the previous huts experiments. The push-pull system in local households showed modest but lower protection against outdoor mosquito landings compared to that obtained during the experimental huts evaluations. This might be due to various reasons: first, the system was affected by the presence and movements of household members outdoors during early hours of night, which may have influenced mosquito densities; secondly, the number of mosquitoes caught during households experiment were lower than the ones collected in experimental huts settings, which limited the statistical power to discriminate effects of push-pull. However, the system significantly reduced mosquito landing and created a diversion effect against *An. arabiensis* mosquitoes, but not against *Culex* mosquitoes.

The lower-than-anticipated protective efficacy of push-pull system seen during both experimental huts and local household settings might also have been due other aspects. While we are unable to clearly identify the main reason, future optimization of the push-pull configurations and the sub-units may identify the critical points for improvement. Nonetheless we hypothesise that the lower efficacy may have been associated with the pull sub-unit (the odour-baited MLB) attracting and not killing large numbers of diverted mosquitoes, perhaps due to lower mosquito population density present in the village during the study period, than in the previous study ^43^. In addition, presence of consistent CO _2_ release from the MLB may have increased numbers of mosquitoes in the area, and as a result reduced observable effect of the push-pull. Further experiments will be required to determine whether the use of additional CO _2_ sources in push-pull systems have negative effects on overall perfomance.

A recent push-pull field study has indicated that there was no additional protection offered by a pull subunit, which was an odour baited device, implying that efficacy of push-pull system is primarily depended on push subunits ^44^. This might have been due to long-range attractants that were used in the MLB, bringing large numbers of mosquitoes close to the households, which were not killed by the device. This indicates that overall communal level protections against mosquito-bites will be seen if the device will be used for the long period of time among many households ^43^. Achieving such gains will require that the number and orientation of push and pull subunits are optimized to increase the efficacy of the system.

## Conclusion

Even with a non-optimised push-pull system set in a peri-domestic area, there was modest protection against early-biting and outdoor-biting *Anopheles arabiensis*, without any increase in indoor mosquito densities. This approach concurrently used two different interventions (lure-and-kill stations and spatial repellents). Low protective efficacy offered by the system against different mosquitoes species suggest a need to do further optimization of the system. Optimal orientation of the subunits, configuration of distance and number of push and pull subunits and dose response studies of the repellent efficacy in a high throughput system such as semi-field system, are some of the characteristics that could be varied to improve efficacy push-pull for malaria prevention. Though we observed no increase in indoor biting risk as a result of push-pull, additional measurements are also needed to be assured that the system is able to offer communal level protection without diverting mosquitoes to non-users both outdoors and indoors. Besides, the variations in protective efficacy of the system between different mosquito species emphasize the need to understand species-specific behavioural responses to spatial repellents and attractants to optimize push-pull systems. Additional experimentation is required to assess how transfluthrin-based products would affect feeding inhibition and mortality of mosquitoes in such push-pull systems. Overall, this approach, if optimised could potentially complement existing malaria interventions even in areas with high pyrethroid resistance.

## Data availability

Data set used to generate these findings are available at Ifakara Health Institute data repository: doi, http://dx.doi.org/10.17890/ihi.2017.10.99

Data are available under the terms of the Creative Commons Attribution International 4.0 license (CC-BY 4.0). The Ifakara Health Institute have provided permission to share the data under all conditions of the CC-BY 4.0 license.
