# Supplementary material for: Small-scale field evaluation of push-pull system against early- and outdoor-biting malaria mosquitoes in an area of high pyrethroid resistance in Tanzania
Source: Wellcome Open Res. 2017 Nov 22;2:112. [Version 1] doi: 10.12688/wellcomeopenres.13006.1 (PMC5840620; doi:10.12688/wellcomeopenres.13006.1)
Supplement: Peer review history

# Peer review: Referee response for version 1

**DOI:** 10.21956/wellcomeopenres.14102.r30795

**Published:** 2018-03-02

**Authors:** Wolfgang R Mukabana

**Affiliations:** 1 School of Biological Sciences, University of Nairobi, Nairobi, Kenya

Mmbando and his colleagues describe a novel push pull system targeting to control early and outdoor-biting malaria mosquitoes in a remote village in rural Tanzania. This paper is one of very few attempts to evaluate such a system on vectors of medical importance. The set up for such work is generally noisier than similar approaches for agricultural pests, yet the group has returned with positive results. Congratulations!

**Major comment**:

 It is important to make clear whom is being protected from mosquito bites in the outdoor environment. By definition this is the protected resource that is being made unattractive or unsuitable to the mosquitoes. The pull and the push sub-units can then be oriented appropriately to evaluate the protective effect of the push-pull system on the outdoor target. In this way we can reach a firmer conclusion on the protective efficacy of push-pull on averting mosquito bites from (unprotected) humans present outdoors. A closer scrutiny of the current set up seems to evaluate the effect of push-pull on protecting indoor sleepers only. The manuscript needs to be revised accordingly if my assessment is correct. I suggest that the authors consult Cook et al. (2007) ^1^ once again.

**Minor comments**:

1. Show Ifakara town in Figure 1.
2. Figure 3 shows that the pull device is located 5m away from the volunteer performing HLC outdoors. However, the main text indicates that the pull device is located 8m away from the volunteer. Please revise accordingly.
3. Show the volunteer located indoors in Figure 3.
4. The abstract mentions that mosquito collections were done from 1830 - 0730 hours but the main text indicates that collections were done from 1800 to 0700 hours. Correct this mistake.
5. Why did the researchers not employ HLC indoors versus HLC outdoors rather than exit traps indoors versus HLC outdoors. Rationalize.

I have read this submission. I believe that I have an appropriate level of expertise to confirm that it is of an acceptable scientific standard.

## References

[1] CookSMKhanZRPickettJA: The use of push-pull strategies in integrated pest management. *Annu Rev Entomol*.2007;52: 10.1146/annurev.ento.52.110405.091407 375-400 10.1146/annurev.ento.52.110405.091407 16968206

# Peer review: Referee response for version 1

**DOI:** 10.21956/wellcomeopenres.14102.r28297

**Published:** 2017-12-04

**Authors:** Joseph M. Wagman

**Affiliations:** 1 Malaria Control and Elimination Program, PATH (Program for Appropriate Technology in Health), Washington, DC, USA

The article describes a set of experimental hut and semi-field experiments that measured the impact of a push-pull system to control outdoor-biting pyrethroid resistant malaria vectors in Tanzania.

 Overall, the work was well designed and executed, and the paper is very well written, concise, and easy to follow. The number of repetitions, the rotating of mosquito collectors and of treatments, the biostatistical rigor, and the fielding of the system in a real-word setting, were all particular strengths of the study. Well done!

 One general comment:

- I think the experimental setup is quite novel in that both the push and pull components are located in the peri-domestic space – a deeper discussion of this novelty, and how results might be compared to other systems that have been reported where the SR is deployed indoors, would be useful. Also, how do these results compare to other push-pull evaluations done with other endo/exophilic vector species from other parts of the world?

 

 Some specific comments include:

- A discussion of why effects of the ‘push’ and ‘pull’ components weren’t tested independently, as well as in combination, would be helpful
- In light of the 0% sporozoite rates observed, some mention of the recorded malaria burden (incidence/prevalence) from Lupiro during the study period
- Actually, could you confirm when the study period was, and what the meteorological/climatological situation was like?
- Some discussion of the baseline comparability (pre-evaluation) of the experimental huts and the collection houses would be helpful
- In the experimental hut setups, why was the use of exit traps advantageous over the use of indoor HLC?
- It might be helpful to clarify the PCR results briefly before the density results, and standardize the use of “ *An. gambiae *complex” and “ *An. arabiensis*” in the tables and discussions
- The mentioning of a diversion effect that was created against *An. arabiensis* in the discussion is somewhat confusing – some clarification of what this refers to would be helpful. The Article describes a set of experimental hut and semi-field experiments that measured the impact of a push-pull system to control outdoor-biting pyrethroid resistant malaria vectors in Tanzania.

I have read this submission. I believe that I have an appropriate level of expertise to confirm that it is of an acceptable scientific standard.
